# DTI-MRI findings in synthetic cannabinoid users

**DOI:** 10.3906/sag-1905-5

**Published:** 2020-06-23

**Authors:** Dilek GÖKHARMAN, Sonay AYDIN, Salih PALTUN, Erdem FATİHOĞLU, Şafak ŞAHİNER, Pınar Nercis KOŞAR

**Affiliations:** 1 Department of Radiology, Ankara Training and Research Hospital, Ankara Turkey; 2 Department of Radiology, Dr. Sami Ulus Training and Research Hospital, Ankara Turkey; 3 Department of Psychiatry, Ankara Numune Training and Research Hospital, Ankara Turkey; 4 Department of Radiology, Faculty of Medicine, Erzincan University, Erzincan Turkey

**Keywords:** Bonzai, synthetic cannabinoids, white matter, diffusion tensor imaging

## Abstract

**Background/aim:**

Synthetic cannabinoids (SCs) are full agonists of both cannabinoid receptors. Conventional magnetic resonance imaging (MRI) findings of SC users are mainly defined as diffusion restriction and T2/FLAIR hyperintensity. Diffusion tensor imaging (DTI) studies examining SC users have shown contradictory results. The aim of this study was to define white matter (WM) changes of SC users using DTI.

**Materials and methods:**

The study included 22 patients with a history of using SC for 5–37 months, and 22 healthy, age and sex-matched control subjects. A total of 41 diffusion gradient directions were used in the acquisition of diffusion imaging data. Fractional anisotropy (FA) and apparent diffusion coefficients (ADC) values were obtained. ROIs were placed on WM areas of normal appearance.

**Results:**

In the SC users, significantly lower FA values were determined in the left temporal lobe (216.2 ± 58.9 vs. 263 ± 27.4; P = 0.002) and right hippocampus (224.5 ± 61.5 vs. 255 ± 24.3; P = 0.040). The ADC values of the hippocampus and temporal lobe were significantly higher than those of the control group on both the left and right sides.

**Conclusion:**

The SC use causes WM microstructural changes, especially in the hippocampus and temporal lobes. DTI is a useful tool to reveal WM changes in SC addicts and can be used earlier than conventional MRI.

## 1. Introduction

Synthetic cannabinoids (SCs) are full agonists of both cannabinoid receptors (CB 1 and CB 2). There are various names to define SCs, such as Bonzai, Jamaika, Aroma, K2, or Kronic. SCs are preferred to original cannabinoid products because they are cheaper, more readily available, and more difficult to detect in drug tests [1,2].

The harmful effects of SCs are similar to those of natural cannabis. As SCs contain various compounds, they can result in greater toxicity than natural cannabis. The most frequent adverse effect to the central nervous system (CNS) is embolic ischemic stroke, similar to that induced by natural cannabis [3].

Conventional magnetic resonance imaging (MRI) findings of SC users are mainly defined as diffusion restriction and T2/FLAIR hyperintensity [4]. Diffusion tensor imaging (DTI) is an MRI technique that is used to assess white matter (WM) microstructure. DTI is an effective tool to assess WM injury by means of quantitatively evaluating fractional anisotropy of brain-water diffusion and the myelin sheath of brain WM fiber bundles. The SC use has been known to reduce the integrity of WM [5]. Fractional anisotropy (FA) is a parameter of DTIthat can be defined as an index for the amount of diffusion asymmetry within a voxel. FA indirectly represents the WM microstructure, and axonal damage or demyelization is associated with reduced FA values [6].

DTI studies examining SC users have shown contradictory results. Some studies have indicated that FA values in SC users increased [7,8], whereas others have reported decreased FA values [9,10]. In addition, these studies have only focused on natural cannabis use. 

The aim of the current study was to define WM changes in the CNS of SC users in comparison with normal controls using DTI.

## 2. Materials and methods

The current study included a total of 22 patients with a history of SC use for 5–37 months and 22 healthy, age and sex-matched control subjects. Any patients with a diagnosis of alcoholism or any addiction other than SC, neurological or psychiatric disorders other than SC addiction, severe hepatic, renal or endocrine disease, and those with a contraindication for MRI examination were excluded from the study. 

The SC users included in the study were also tested using the Montreal cognitive assessment (MoCA) test [11] to define cognitive functions and any possible relationship between cognitive impairments and DTI findings. 

MRI examinations were performed with a 1.5 Tesla MRI system (Magnetom Aera, Siemens Healthcare GmbH, Erlangen, Germany). The diffusion imaging data were acquired from 41 diffusion gradient directions (b-value = 1000 s/mm2) and T2 and FLAIR reference images were also obtained (repetition time = 6500 ms, echo time = 90 ms, voxel size=1 ×1 ×2 mm3). The DTI protocol involved structural 3D-T1, T2-weighted images.

FA maps were created on a Siemens, Leonardo workstation using the Neuro3D application, and FA values were then measured on the FA maps. ADC values were obtained using the same software. 

**Figure 1:**
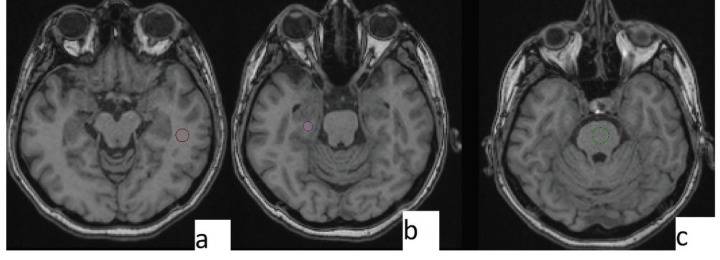
Measurement levels. Temporal lobe (a), hippocampus (b), and brainstem (c).

FA and apparent diffusion coefficients (ADC) values were acquired using approximately 30 mm2 regions of interest (ROI). ROIs were placed on WM areas of normal appearance if any WM lesions were present on T2 or FLAIR images. All measurements were performed by the same researcher (SA). Three ROIs were placed into the examined location, and the mean values of 3 measurements were recorded as the final data. Measurements were performed from temporal lobes, the hippocampus, and the brainstem since SCs have been known to affect the limbic system and, in addition, previously performed DTI studies generally chose the same locations (5). The brainstem was also preferred in the investigation of life-threatening effects. Measurement levels can be seen in Figure 1.Approval for the study was granted by the Institutional Ethics Review Board, and informed consent was obtained from each participant.

### 2.1. Statistical analysis

Data obtained in the study were analyzed statistically using SPSS statistics 21.0 (IBM Corp., Armonk, NY, USA). Normal distribution was tested with the Kolmogorov-Smirnov test. Continuous parameters were stated as mean ± standard deviation (SD), and numerical variables without normal distribution were shown as median (minimum-maximum) values. Normally distributed variables were compared between the patients and control groups with the Student’s t-test, whereas parameters with abnormal distribution were compared via the Mann-Whitney U test between the 2 groups. The correlations between age, sex, duration of usage, and FA values were evaluated with the Pearson and Spearman correlation tests.A two-tailed value of P < 0.05 was considered statistically significant.

## 3. Results

All of the participants were male, and all were currently using SC. Both the SC users and control subjects smoked at least one pack of cigarettes a day. The mean age of the SC users and control group were 23.3 ± 5.6 years and 22.7±4.8 years, respectively. Median duration of SC use was 13 (5–37) months, and median daily SC use was 4 (1–12) times per day. 

The median MoCA test result of the SC users was 27; only 3 SC users had an abnormal test result (18,18, and 20 points, respectively, with ≤21 points defined as abnormal). 

Conventional MRIs of the patients were mostly normal. Subcentimeter WM lesions were detected in only 4 patients. The lesions were located at the temporal (3 patients) and parietal (1 patient) lobes.

**Figure 2:**
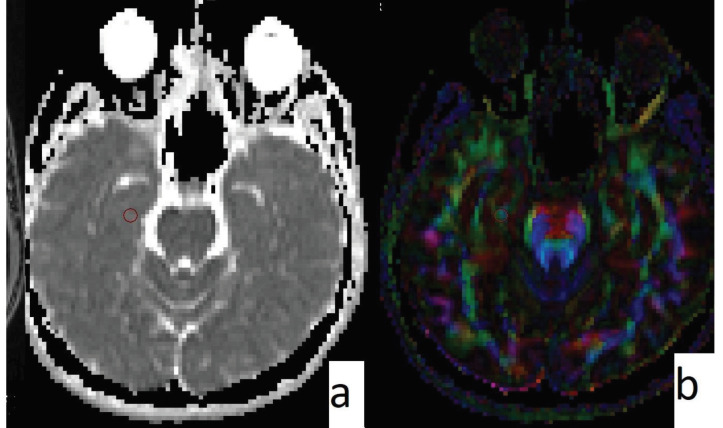
Male, 22 years old, SC addict. ADC map (a) and FA map (b) can be seen. ROI was placed on the right hippocampus. ADC value is 893; FA value is 231 (significantly lower than the control group).

**Figure 3:**
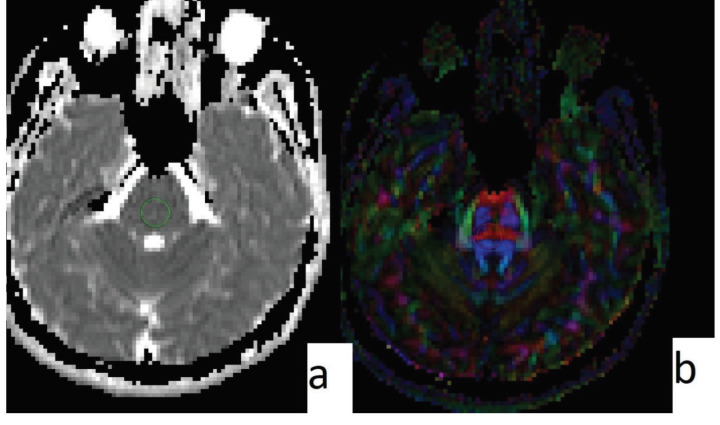
Male, 32 years old, control group. ADC map (a) and FA map (b) can be seen. ROI is placed on the brainstem. ADC value is 741; FA value is 440 (similar to the control group).

Significantly lower FA values were obtained from the left temporal lobe (216.2 ± 58.9 vs. 263 ± 27.4; P = 0.002) and right hippocampus (224.5 ± 61.5 vs. 255 ± 24.3; P = 0.040) in the SC group than from the healthy control group (Figure 2). The FA values obtained from the brainstem were similar in the SC users and the control group (435.6 ± 36.5 vs. 442.1 ± 62.2) (Table 1) (Figure 3).

**Table 1:** Significantly different FA values.

	SC users	Control group	P-value
Left temporal lobe	216.2 ± 58.9	263 ± 27.4	0.002
Right hippocampus	224.5 ± 61.5	255 ± 24.3	0.040

The ADC values acquired from the brainstem did not differ between the SC users and the control group (703.3 ± 145.2 vs. 752.7 ± 24.2). In the SC group, the ADC values of the hippocampus and temporal lobe were significantly higher than those of the control group for both the left and right sides (right temporal: 824.5 ± 41.6 vs. 791.5 ± 16.4; P = 0.002; left temporal: 838 ± 28.5 vs. 807.8 ± 38.4; P = 0.005; right hippocampus: 849.2 ± 87.8 vs. 792.6 ± 20.1; P =0.007; left hippocampus: 859 ± 116.2 vs. 803.2 ± 25.9; P = 0.038) (Table 2). No significant correlation was determined between the ADC and FA values and age, duration of usage, and amount of daily use. No significant correlation was detected between the MoCA results and FA and ADC values. The number of patients with an abnormal MoCA result (3 patients) was insufficient for statistical analysis to be applied. 

**Table 2:** Significantly different ADC values.

	SC users	Control group	P-value
Right temporal lobe	824.5 ± 41.6	791.5 ± 16.4	0.02
Left temporal lobe	838 ± 28.5	807.8 ± 38.4	0.005
Right hippocampus	849.2 ± 87.8	792.6 ± 20.1	0.007
Left hippocampus	859 ± 116.2	803.2 ± 25,9	0.038

## 4. Discussion

The main aim of the current study was to define WM changes resulting from SC use. The results showed that SC addiction causes WM structural changes, and these changes can be revealed with DTI. 

Cannabinoids are widely used throughout the world. The main psychoactive compound of cannabinoids is tetrahydrocannabinol, which can activate cannabinoid receptors in the brain. Cannabinoid receptors are mostly located in basal ganglia, the cerebellum, and limbic cortices. SCs are cannabinoid-like substances that are widely used because of their cheap costs and easy availability [12]. In Turkey, the most popular SC is Bonzai, and its chemical compound mainly consists of JWH-081 [13]. 

There is limited information in the literature about SC toxicity on the CNS, and the studies thathave been published are generally case reports. The toxic findings defined in the literature are related to the combination of direct neurotoxicity and cerebral hypoxia, as in natural cannabinoids. Therefore, MRI findings generally present ischemic findings [14,15]. In a case report about JWH-081 toxicity, it was stated that JWH-081 might cause myelin toxicity and leukoencephalopathy, although the report only contained conventional MRI data [4]. 

It has been emphasized in the literature that myelin-related genes, myelin basic proteins, and myelin proteolipid proteins are altered following prolonged exposure to cannabis [16]. Chronic SC exposure has also been shown to cause dose-dependent downregulation of CB1 receptors in an animal model (rodents), especially in the hypothalamus and hippocampus. This downregulation might cause apoptosis of oligodendrocytes, the myelinating cells in the CNS, and thereby deplete myelinization [17,18]. Consistent with this information, the current study results demonstrated that the WM of SC users is changed, especially in the hippocampus and temporal lobe since the FA values obtained from these areas were lower than those of the control group. In a similar study [5], it was shown that SC use decreases FA values obtained from the left temporal lobe, similar to the current study results. However, unlike the current study’s results, the previous study found that SC use had an effect on the left hippocampus. This difference could be the result of the duration of SC use. In the abovementioned study [5], all of the participants had been using SC for 30 ± 14.4 months (mean value); our population had a median duration of use of 15 months. Therefore, there is a need for further prospective studies to be able to clarify the order and process of influence on specific CNS regions. It is also highly possible that both sides of the brain can be similarly affectedwhen the above-mentioned pathogenesis [17,18] is considered. 

The results of the current study showed that FA values obtained from the left temporal lobes of SC users were significantly lower than those of the healthy control group. These findings are consistent with the literature. Recently performed studies have similarly stated that FA values are reduced in the temporal lobes of cannabis users [19,20] and in the left temporal lobe of SC users [5]. 

No significant FA and ADC value changes in the brainstem were determined between SC users and the control group, and these results were consistent with the literature. It has been stated in previous studies that CB1 receptors are expressed at a low level in the brainstem [21], so the effect of SC on the brainstem is weak. 

The ADC values were also significantly higher in both temporal lobes and the hippocampus of SC users in the current study. The ADC values are also related with WM degeneration, and this was similar to the FA values. It has been stated that in normal aging or in degenerative processes, ADC generally increases and FA decreases [22,23]. The current study results of FA and ADC are both consistent with the literature and support the effectiveness of DTI in the detection of degenerative WM changes in SC users. 

No significant correlation was determined between FA values and duration of use and amount of daily use, contrary to expectations. To the best of our knowledge, there is no study in the literature that has examined these relationships. One possible explanation of these results could be that downregulation of CB1 receptors occurs earlier than after 5 months of SC use and is not dose-dependent. However, it is also possible that the current study population was not large enough to detect such a correlation. Further prospective studies are needed to clarify these possible relationships. 

Some limitations of the study must be emphasized. The sample size was relatively small as it is difficult to find patients addicted to only SC and who are also willing to participate in a study of this kind. The participants in this study were using Bonzai, a popular SC in Turkey, the content of which is known to be mainly JWH-018. However, it was impossible to detect the exact content and ratio of contents of the Bonzai used by the participants. The study population comprised of males only, so the results cannot be generalized to the female population. Multiple addictions and psychiatric problems, together with addiction, are also common problems. Addicts using SC only were selected from their psychiatric reports and statements. However, it was not possible to create a fully homogenous study group. Since all of the measurements were only performed by the same researcher one time, we cannot offer inter/intraobserver variability data. 

In conclusion, SC use causes WM microstructural changes, especially in the hippocampus and temporal lobes. These changes can be detected via FA and ADC values using DTI. Therefore, DTI is a more useful tool than conventional MRI in the early detection ofWM changes in SC addicts.

## Disclaimer

No funding was received for this study.

## Conflict of interest

All authors declare that they have no conflict of interest. 

## Informed consent

All procedures performed in the following studies involving human participants were in accordance with the ethical standards of the institutional and/or national research committee and within the standards set in the 1964 Helsinki Declaration and its later amendments or comparable ethical standards.Institutional review board approval was obtained, and the need for informed consent was waived for the current retrospective study.

## References

[ref1] (2013). Patterns of synthetic cannabinoid use in Australia. Drug and Alcohol Review.

[ref2] (2013). Spicing things up: synthetic cannabinoids. Psychopharmacology.

[ref3] (2011). Cannabis use, ischemic stroke, and multifocal intracranial vasoconstriction: a prospective study in 48 consecutive young patients. Stroke.

[ref4] (2017). Buzz juice: neurological sequelae of synthetic cannabinoids. Journal of Clinical Neuroscience: Official Journal of the Neurosurgical Society of Australasia.

[ref5] (2016). Abnormal white matter integrity in synthetic cannabinoid users. European Neuropsychopharmacology.

[ref6] (2002). Diffusion tensor imaging and its application to neuropsychiatric disorders. Harvard Review of Psychiatry.

[ref7] (2006). Brown. Harm Reduction Journal.

[ref8] (2014). Long-term effects of marijuana use on the brain. Proceedings of the National Academy of Sciences of the United States of America.

[ref9] (2014). Worth the wait: effects of age of onset of marijuana use on white matter and impulsivity. Psychopharmacology.

[ref10] (2010). White-matter abnormalities in adolescents with long-term inhalant and cannabis use: a diffusion magnetic resonance imaging study. Journal of Psychiatry & Neuroscience: JPN.

[ref11] (2005). The Montreal cognitive assessment, MoCA: a brief screening tool for mild cognitive impairment. Journal of the American Geriatrics Society.

[ref12] (2010). Addictive illegal drugs: structural neuroimaging. AJNR American Journal of Neuroradiology.

[ref13] (2013). Review of detection frequency and type of synthetic cannabinoids in herbal compounds analyzed by Istanbul Narcotic Department of the Council of Forensic Medicine, Turkey. Journal of Forensic and Legal Medicine.

[ref14] (2012). Spice drugs are more than harmless herbal blends: a review of the pharmacology and toxicology of synthetic cannabinoids. Progress in Neuro-Psychopharmacology & Biological Psychiatry.

[ref15] (2015). Tetrahydrocannabinol induces brain mitochondrial respiratory chain dysfunction and increases oxidative stress: a potential mechanism involved in cannabis-related stroke. BioMed Research International.

[ref16] (2002). Assessment of cannabinoid induced gene changes: tolerance and neuroprotection. Chemistry and Physics of Lipids.

[ref17] (2010). effects on CB1 receptor density in the adolescent brain: an autoradiographic study using the synthetic cannabinoid HU210. Synapse.

[ref18] (2002). Cannabinoids promote oligodendrocyte progenitor survival: involvement of cannabinoid receptors and phosphatidylinositol-3 kinase/Akt signaling. The Journal of Neuroscience: the Official Journal of the Society for Neuroscience.

[ref19] (2014). White matter abnormalities and cognitive impairment in early-onset schizophrenia-spectrum disorders. Journal of the American Academy of Child and Adolescent Psychiatry.

[ref20] (2015). White matter fractional anisotropy over two time points in early onset schizophrenia and adolescent cannabis use disorder: a naturalistic diffusion tensor imaging study. Psychiatry Research.

[ref21] (2002). Classification of cannabinoid receptors. International Union of Pharmacology.

[ref22] (2010). Age-related regional variations of the corpus callosum identified by diffusion tensor tractography. Neuro Image.

